# Has evidence‐based medicine ever been modern? A Latour‐inspired understanding of a changing EBM

**DOI:** 10.1111/jep.12752

**Published:** 2017-05-16

**Authors:** Sietse Wieringa, Eivind Engebretsen, Kristin Heggen, Trish Greenhalgh

**Affiliations:** ^1^ Medical Faculty, Institute of Health and Society University of Oslo Oslo Norway; ^2^ Department of Continuing Education/Evidence‐based Health Care University of Oxford Oxford UK; ^3^ Medical Faculty, Primary Care Health Sciences Oxford University of Oxford Oxford UK

**Keywords:** epistemology, evidence‐based medicine, health care, medical research, philosophy of medicine

## Abstract

Evidence‐based health care (EBHC), previously evidence‐based medicine (EBM), is considered by many to have modernized health care and brought it from an authority‐based past to a more rationalist, scientific grounding. But recent concerns and criticisms pose serious challenges and urge us to look at the fundamentals of a changing EBHC.

In this paper, we present French philosopher Bruno Latour's vision on modernity as a framework to discuss current changes in the discourse on EBHC/EBM. Drawing on Latour's work, we argue that the early EBM movement had a strong modernist agenda with an aim to “purify” clinical reality into a dichotomy of objective “evidence” from nature and subjective “preferences” from human society and culture.

However, we argue that this shift has proved impossible to achieve in reality. Several recent developments appear to point to a demise of purified evidence in the EBHC discourse and a growing recognition—albeit implicit and undertheorized—that evidence in clinical decision making is relentlessly situated and contextual. The unique, individual patient, not abstracted truths from distant research studies, must be the starting point for clinical practice. It follows that the EBHC community needs to reconsider the assumption that science should be abstracted from culture and acknowledge that knowledge from human culture and nature both need translation and interpretation. The implications for clinical reasoning are far reaching. We offer some preliminary principles for conceptualizing EBHC as a “situated practice” rather than as a sequence of research‐driven abstract decisions.

## 1 INTRODUCTION

The evidence‐based movement is over 25 years old and has profoundly affected the way health care is practised. Originally known as evidence‐based medicine (EBM) and now usually referred to as evidence‐based health care (EBHC) to embrace all health care professions, the movement has achieved much but also drawn criticism and concerns.

For instance, in 2014, a group of scholars questioned whether the EBHC movement was “in crisis” as a result of a number of unintended consequences of its earlier success^1^:

the evidence based ‘quality mark’ has been misappropriated by vested interests; the volume of evidence, especially clinical guidelines, has become unmanageable; statistically significant benefits may be marginal in clinical practice; inflexible rules and technology driven prompts may produce care that is management driven rather than patient centred; and evidence based guidelines often map poorly to complex multimorbidity.

Many authors consider that the emergence of EBM/EBHC (a movement that continues to evolve) marks the shift from the traditional “priestly authority” of doctors to a more modern, knowledge‐based—and hence rational and scientific—authority.^2^, ^3^, ^4^ As such, EBM/EBHC deserves analysis within the wider context of modernity in general. In this paper, we consider the discourse on EBM/EBHC and its future in the light of work by the French anthropologist and philosopher Bruno Latour, who analysed the emergence of science and technology in late modernity in one of his classic books *We have never been modern*.^5^ In the next section, we summarize relevant ideas from this book and apply them to EBM/EBHC.

### 1.1 “We have never been modern”

The term “modernity” is generally used to describe a historic period in European arts, politics, science, and the humanities that began in the 16th century with the rise of the new cosmology and experimental science and extended to the 20th century. It conveys the idea of a general progress towards the rejection of traditional and religious explanations as envisioned by enlightenment philosophers as well as early social theorists such as Max Weber, Emile Durkheim, and Auguste Comte. Their work culminated in a broad range of related theories and ideas still prevalent today. With modernity came increasing secularization, rational thought and rejection of tradition along with the firm belief that reality is based on empirically testable scientific laws.

Bruno Latour wrote several classic books on the practices and nature of science that already inspired others to discuss relevant issues of the evidence‐based movement, such as the standardization of morals and patients^6^ and the implementation of innovations.^7^

**Figure 1:**
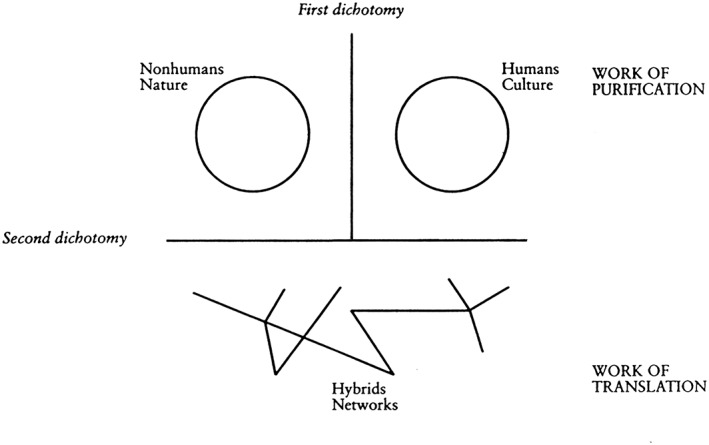
Purification and translation

In *We have never been modern*, Latour acknowledges many versions of modernism, which is recognizable when society is engaged in 2 separate practices: purification, which means to make a distinction between nature (which has always been) and human culture (which is man made), and translation/hybidrization, defined as efforts to combine elements of nature and culture in “hybrids networks” (Figure 1).

Latour gives as an example of translation a debate on climate change, in which translation efforts seek to align elements of both nature and culture—specifically, “[the] chemistry of the upper atmosphere, scientific and industrial strategies, the preoccupations of heads of state, [and] the anxieties of ecologists.”

Latour argues that modernists recognize that nature and culture have interconnections, but they seek to make them analytically separable. In particular, say modernists, science should be politics free. Modernists' assumption (what Latour calls “the modernist constitution”) entails that nature is always objectively knowable and never man made and human culture is always made up and never influenced by nature.

According to this view, we would be “modern” when and to the extent that we could successfully separate nature and culture and subject the former to rational scientific analysis. However, argues Latour, despite the application of advanced scientific techniques and technological apparatus, modern society is struggling and failing to keep these 2 phenomena apart. Nature and culture remain inextricably linked, just as they were in the premodern era.

To resolve this modernist problem, Latour does not argue for a return to premodern obscurantism, ie, deliberately precluding full understanding of anything. Instead, he introduces an alternative take on reality, which he calls a “middle kingdom.” In this view, the world, consisting of both nature and culture, is explained with reference to “hybrids” or the so‐called “quasi‐objects.” He offers a number of examples including soccer balls,^8^ frozen embryos, expert systems, digital machines, sensor‐equipped robots, hybrid corn, data banks, and psychotropic drugs.^5^ All of them are naturally real in the sense that they obey physical laws, but are also culturally real in the sense that they have cultural meaning and social significance. They are objects and nonobjective concepts at the same time. According to Latour, moderns deny quasi‐objects even though they depend on them. To enter the middle kingdom means to acknowledge that nature and culture can never be kept apart and that the modern project is equally dependent on purification and translation.

### 1.2 Early EBM as a modernist movement

The emergence of EBM in the early 1990s can be understood as a modernist movement. For instance, an article by the EBM Working Group in JAMA in 1992 opens as follows:

A new paradigm is emerging. EBM de‐emphasises intuition, unsystematic clinical experience, and pathophysiologic rationale as sufficient grounds for clinical decision making and stresses the examination of evidence from clinical research.^9^

In the above quote, real patients and conditions are understood as hybrids of 2 pure forms: objective nature and subjective culture. Clinical research is depicted as able to provide “facts” of biology and epidemiology, as far as possible void of human intuition or experience. Indeed, the programme of training that was to be offered to the early students consisted (more or less) of a menu of tools, techniques, and processes for separating objective (hence, trustworthy) evidence from subjective (untrustworthy) opinions, biases, and perspectives. Thus, EBM began with explicit aspirations to modernism that still persevere today.^10^ For instance, in a paper on evaluating e‐health interventions, the authors write:

[…] health information systems should be evaluated with the same rigour as a new drug or treatment programme, otherwise decisions about future deployments of ICT in the health sector may be determined by social, economic, and/or political circumstances, rather than by robust scientific evidence.^11^

Not all early protagonists of EBM rejected the value of clinical expertise. Indeed, Sackett et al famously defined EBM as an approach that integrates the best external evidence with individual clinical expertise and patients' choice.^12^ But even this, more accommodating definition remains essentially modernist. The fundamental issue here is the concept of “the best external evidence.” Although Sackett et al advise practitioners to “integrate” clinical expertise and patient choice (which implies some kind of hybridization), they persist in conceptualizing evidence in the form of general truths that are external to the messiness of the clinical encounter and arrived at through rational, objective means (notably, randomized controlled trials [RCTs]).

Compare with Latour who writes:

The moderns […] did not make quasi‐objects disappear by eradication and denial, as if they wanted to simply repress them. On the contrary, they recognized their existence but emptied it of any relevance […] The modern explanations consisted in splitting the mixtures apart in order to extract from them what came from the subject (or the social) and what came from the object. Next they multiplied the intermediaries in order to reconstruct the unity they had broken and wanted none the less to retrieve through blends of pure forms.

Thus, while Sackett and other early leaders of the EBM movement celebrated clinical expertise and the patent voice, they nevertheless insisted that evidence is developed in a secluded space outside the clinical encounter and only then translated through integration with expertise and preferences. In this way they reproduced the dichotomy between science and translation typical of modernism.

By the late 1990s, critical social scientists were reflecting that EBM had succeeded in producing

…[a] Cartesian epistemology of differentiation—a reality of distinct subjects and objects—in which research evidence and clinical practice are seen as dualistically opposed. Evidence, in this context, is seen as a commodity, whose better, quicker, easier to access, and increasingly electronic transfer, from the research pole to that of practice will lead to the more effective clinical management of patients […]. Whilst the appeal of this dualist model is its maintenance of stable boundaries and firm divisions: objective/subjective, research/practice, facts/values, such thinking is almost always structured so as to privilege one side of the dualism over the other. This has made possible the hierarchical distinction between, for example, the objective ‘facts’ of biomedical research and the subjective ‘mere knowledge’ of clinical practice. Here, an analytic and disembodied (scientific) organisation of knowledge is privileged over more tacit and situated experiences: a body of evidence, separated from its social context, that can be unilaterally transmitted from the research setting—where it is known—to the world of practice—where it is not.^7^

The 1990s and 2000s saw an exponential expansion in the kind of research designated by the EBM community as “robust”—defined in terms of the methods used to reduce or eliminate bias in the collection and analysis of empirical data. The large RCT became the bread‐and‐butter type of research and in its trail rapidly followed increasingly sophisticated systematic reviews and meta‐analysis, instruments to appraise clinical practice guideline development, frameworks to guide and assess clinical care pathways, and the institutionalization of implementation as a science.

### 1.3 The “movement in crisis”: EBM's receding modernist vision

The original vision of the EBM movement was that within a generation, the evidence base for clinical decisions would have been collected, collated, and distributed for easy access by clinicians and policymakers. This knowledge base—“best evidence”—would be objectively verifiable and readily updated in real time as new research evidence from robust study designs accumulated.

However, research evidence in many fields remains heavily contested. The scientists, clinicians, and guideline developers involved are usually EBM experts; they understand the rules for interpreting clinical trials and screening programmes perfectly well. Yet they each interpret the evidence differently. These disagreements between experts often originate not from the data but from a difference in values.^13^ Principles like confidentiality, equality, and preventing harm affect how “facts” are understood. Reinterpretation of existing evidence in national breast cancer screening programmes based on mammography caused heavy debate about their effectiveness and the risks of overdiagnosis.^14^ The discussion on the harms and benefits of statins in low‐risk patients and the elderly is fierce and ongoing as some trialists and systematic reviewers are accused of conflict of interests.^15^ And the medical community was shocked when recommendations for Tamiflu^16^ and paroxetine in adolescence^17^ were reverted after previously unpublished research data were disclosed and reinterpreted by others. Again and again, evidence‐based value‐free “facts” turn out to be value laden (and for good reason).

In the light of these examples, the warning offered in 1997 by Haridimos Tsoukas was remarkably prescient (although few in the EBM movement heeded it at the time):

Contrary to how knowledge was viewed in pre‐modern societies, knowledge now tends to be understood as information, that is as consisting of objectified, commodified, abstract, decontextualized representations. The overabundance of information in late modernity makes the information society full of temptations. It tempts us into thinking that knowledge‐as‐information is objective and exists independently of human beings; that everything can be reduced into information; and that generating ever more amounts of information will increase the transparency of society and, thus, lead to the rational management of social problems. However, … the information society is riddled with paradoxes that prevent it from satisfying the temptations it creates. More information may lead to less understanding; more information may undermine trust; and more information may make society less rationally governable.^18^

The original goals of EBM to provide a methodology to generate independent truths and objectively assess the certainty of facts have so far proved elusive. Society's influence pervades. Despite the hopes and predictions of its forefathers, EBM has never become modern.

### 1.4 EBHC in 2017—moving towards a “middle kingdom?”

A number of recent developments suggest that the EBM/EBHC movement is gradually recognizing its rigid adherence to modernism while it tempts to get to grips with its stance towards nature on one side and human society on the other.

1. The discourse on EBHC displays a greater degree of reflexivity from within the community. Whereas the early protagonists of EBM saw the movement as part of the inevitable march of scientific progress, social scientists have always viewed EBM as historically—and politically—situated. Judith Greene,^19^ for instance, notes that:

The EBHC movement has attracted critical comments from sociologist and policy analysts from a number of perspectives: that for instance, clinical epidemiology is not always the most appropriate framework for decision making in healthcare […] and that EBM can be deconstructed as a discourse which functions to maintain the ‘purity’ of medical practice in the face of threats to autonomy […]. [O]ne key problem within the EBM movement lies in its assumption of rationalistic behaviour and linear change: that evidence is stable and independent of social relationships.

But now the EBHC community too appears to embrace these criticisms as it tries to find solutions for the inability to fully be modern. For example, to deal with vested interests, the ALLTrials campaign^20^, ^21^ calls for better registration of planned trials, summary results reported within a year after the end of all trials, and publication of a full report including all harms. These may still be considered modernist, purifying activities. But the practices of the initiative, creating momentum, networking, lobbying, and beating the pharmaceutical industry at its game, are not. They make evidence‐based–minded clinicians, researchers, and guideline developers more and more aware that medical facts are not so objective as they seem. They turn out to be constructed, situated within specific agendas and alignments of key players in human society.

- The EBM/EBHC no longer views the result of a sample mean in an RCT as applicable unproblematically to the single case scenario. There is a more sophisticated understanding of the translational process from scientific evidence to a particular situation such as a complex intervention at the organizational and system level or an individual patient in a particular situation.

For example, inspired by the concepts of “shared decision making”^22^ and “person‐centred care,”^23^ guideline makers are supporting individualized clinical decisions by providing new tools in new formats, as, for instance, the Magic app^24^ or Option grids.^25^

What is new about these instruments is that they do not just prescribe what objective, purified nature says (does the drug work?), but also present arguments from human culture (Do patients value it? Is it affordable?). True, as they still separate nature and human society in neat categories, they remain largely modernist. But nature and society are put on a more equal footing than ever before.

These developments are still somewhat removed from what it takes to care for an actual patient. That would be what philosopher Annemarie Mol calls a “logic of care”—a constant tinkering, while avoiding neglect^26^ or a relationship‐based care—following the story of the person in context, in the words of Schei^27^:

a relational competence, where empathic perceptiveness and creativity render doctors capable of using their personal qualities, together with the scientific and technologic tools of medicine, to provide individualized help attuned to the particular circumstances of the patient.

But they do reveal a growing interest in the EBHC discourse to start thinking from the standpoint of real patients in real encounters as with Latours quasi‐objects.

- There is an increasing interest in less purified kinds of knowledge, research methods, and styles of reasoning.^28^

For example, works by Gigerenzer^29^ on heuristics and other researchers on gut feelings^30^ provide evidence that decisions based on not‐so‐explicit human reasoning and inferences do give good, and sometimes even better, outcomes in reality. Ethnographic research by Gabbay and Le May and observational research by Zwolsman ea^31^, ^32^ showed that in everyday practice, most clinical decisions commonly rely on tacit, hard‐to‐explain knowledge, influenced by past experiences, peers, and contexts. Pragmatic randomized trials have been designed to test interventions in the actual context of where they will be used.^33^ And guideline makers urge that in practice, good recommendation requires more kinds of knowledge than just RCTs.^34^

These examples show an understanding that the products of modernist practice of purification (resulting in explicit law like rules of medicine) need to be watered down to become applicable “in reality.” Latour writes:

We do not need to attach our explanations to the two pure forms known as the Object or Subject/Society, because these are, on the contrary, partial and purified results of the central practice that is our sole concern. The explanations we seek will indeed obtain Nature and Society, but only as a final outcome, not as a beginning.^5^

Purified findings from well‐designed RCTs and reviews are wonderful insights, but at the end of the day, they may prove useless in the face of an actual patient, in the here and now.

Of course, consultations themselves have always been about translation and hybridization. In consultations, patients and health care professionals swiftly connect molecules, plaques, platelets, cells, average blood pressures, drugs, lifestyle, food, function, meaning, values, sense, and care. What is new is the growing acknowledgement in the discourse on EBHC that these are not clearly separable in objective nature or subjective culture (as critical social scientists have argued for decades).

## 2 DISCUSSION

In his book, Latour sought to eradicate the divide between science and culture. His middle kingdom implies a fundamental questioning of the modernist conception of science that extends beyond EBM.

While current initiatives in the EBHC movement may concede the existence of a middle kingdom—that is, they accept that research evidence cannot sit above history, culture, or politics but is recursively linked^1^ to all 3—there remains an important paradox. As Latour argued about science in general in *We have never been modern*, EBHC cannot acknowledge this middle kingdom without ceasing to be modern and collapsing back into premodernism.

This creates an ambivalence: On the one hand, EBHC (at least as it has developed) depends on mediation, translation, or a “cultural supplement.” On the other hand, it still relies on the purification of science to be separate from, and more objective than, real‐world messiness. The whole concept of “best evidence” hinges upon such a divide. If not, it risks becoming “unscientific.” Inevitably, then, EBHC must move towards a middle kingdom to deal with the paradoxes it has already generated (such as the dominance of vested interests in the publication of large RCTs in mainstream academic journals). But EBHC can never fully embrace this middle kingdom without effectively rejecting the very concept of evidence on which its central claims are based. Instead of resolving this paradox, the EBHC movement must coexist with it and “muddle through.”

Latour argues that while modernity is built on a myth (the separability of nature and culture), it nevertheless produces benefits. Modernity generates the need for translation, hybridization, and cultural/technological supplements, a need that follows from the act of purification itself. The practice of purification also leads very efficiently to many new insights (smoking leads to cancer), concepts (statistical significance), and natural objects (DNA). All these quasi‐objects would not have existed in a premodern society, he argues. Indeed, it is hard to underestimate the impact EBHC has had with its renewed methods of induction as means to purify clinical reality into more or less stable rules and recommendations.

We contend that any new interpretation of EBHC should not seek to refute the fundamental principles of clinical epidemiology or the position of the RCT as the best study design for reducing bias in human experiments. We are not arguing for a return to the priestly authority of doctors, to anecdotal evidence, or to guideline development by GOBSAT (“good old boys sat around a table”). Rather, we suggest that a progressive, mature, “middle kingdom” EBHC would reframe the concepts of “evidence” and “medicine/health care” in EBM/EBHC in several different ways:

- The new EBHC should reflexively reject the science‐culture dichotomy. Especially in some circumstances (for example, where different stakeholder groups hold widely different values and priorities; where vested interests loom large; or where uncertainty is inherent^35^), “pure” evidence is unlikely to be obtained by stripping away real‐world messiness or imposing rigid or unworkable protocols. As Contandriopoulos points out:

Collective knowledge exchange and use are phenomena so deeply embeded in organizational, policy, and institutional contexts that externally valid evidence pertaining to the efficacy of specific knowledge exchange strategies is unlikely to be forthcoming.^36^

Rather, a mature EBHC will seek to incorporate the messiness of real‐world hybridization into a wider range of approaches and methodologies. Concepts of a “gold standard” and a “hierarchy of evidence” become highly problematic in such circumstances. Randomized controlled trials require “supplements” to create better inferences for a complex reality, be it pragmatic trials, pattern recognition modelling, participatory codesign, or other approaches.

- The new EBHC recognizes that all evidence—whether derived from science on the one hand or from human culture (patient preferences, opinions, insights, etc) on the other—requires translation and interpretation. This is in contrast to a recent study where researchers found that guideline panel members differentiated between “objective evidence” from studies and “insights” from patients. They write: “There is a tendency to try to weigh‐up the patients' views *against* the clinical and economic data, which proves difficult.”^37^ With Latour's framework, it becomes clear why nature and society are hard to separate. If we accept the legitimacy of the middle kingdom, we must accept that research evidence cannot be placed on a pedestal apart from other evidence in this way, as well as accepting that research evidence will always be developed outside the relationship‐based care for the patient and based on a logic, which is different from the relational logic inherent in a clinical encounter.

The danger of the dominant discourse of rationality is that by marginalising and devaluing the role of individual judgement, it undermines rather than strengthens actors' capacity to act.^38^

- The new EBHC acknowledges the need to reason not only from the individual to the general or from the general to the individual but from individual to individual (casuistry^39^). In the old EBM, health care needs to be purified to find its (assumed stable) laws of nature. For instance, people with hypertension have a higher cardiovascular risk. We, the society of humans, make mistakes against the absolute rules of nature, which are typically referred to as “cognitive biases.” Instead, Latour urges us to reason from the original “quasi‐object/hybrid” that is the patient in practice in clinical context. The question then becomes as follows: Is this patient with hypertension one of the group of patients that would form the “rule” (hypertension causes increased cardiovascular morbidity)? It is a small difference, but crucial in EBHC: It forces clinicians to challenge the rule with every patient they see, to ask if there actually is a rule or an original new situation, and to recognize that the rule found in a certain reference population (eg, incorporated into a guideline) cannot remain a law forever. It will change as the original/real patients in their contexts are changing. It is about causality departing from a dynamic world context, not a stable controlled laboratory environment.^40^
- The new EBHC recognizes its own limitations in relation to decision support and inductive inference in health care.

Latour's framework unveils EBHC as just one of the activities (practices) of everyday health care. It is the practice of purification in that realm: making inferences to produce new insights and good decisions.

But as a result of the focus on purification practices of early EBM, the purpose of health care itself became skewed towards aiming for objective evidence from nature at the expense of care and equity. The concept of health care as caring, loving, and nurturing has largely been lost in the process.^41^

This was not intended. For Archie Cochrane, who the early EBM proponents often cited in support of their view, health care had 2 equality important dimensions:

I see the NHS, rather crudely, as supplying on the one hand therapy, and on the other board and lodging and tender, loving, care.^42^

Purification and hybridization have their place, but not predominantly. Health care is about making good inferences (which is the role of EBHC), about equity, and about relationship‐based, loving care. Paraphrasing Latour, health care should once again be reinstated as broad concept that has both “science and society as its satellites.”

## 3 FINAL REMARKS

In this paper, we aimed to provide an understanding of the current position changes and the future of the evidence‐based movement by introducing Latour's views on modernity as a framework for discussion. An ongoing dialogue within the movement is needed about the role, the paradox, and benefits and harms of the modernist practice of purification. With Wood, we would argue for more interdisciplinary practices: “innovation is neither natural or inevitable, but constantly negotiated and aligned—a path forged within an assemblage of scientific and organisational and behavioural factors.”^7^ Rather than a sequence of research‐driven abstract decisions, EBHC should be conceptualized as a situated practice, always starting from the real problems of individual real patients (and real organizations and systems) in their real contexts.

## References

[1] Greenhalgh T , Howick J , Maskrey N . Evidence based medicine: a movement in crisis? BMJ Br Med J. 2014;348:g3725. 24927763 10.1136/bmj.g3725 PMC4056639

[2] Glasziou P , Del MC , Salisbury J . Evidence Based Medicine Workbook. BMJ Publishing Group; 2003.

[3] Timmermans S , Berg M . The Gold Standard. Philadelphia: Temple University Press; 2003.

[4] Howick JH . The Philosophy of Evidence‐based Medicine. BMJ Books; 2011.

[5] Latour B . We Have Never Been Modern. Cambridge: Harvard University Press; 1993.

[6] Valkenburg G , Achterhuis H , Nijhof A . Fundamental shortcomings of evidence‐based medicine. J Health Organ Manag. 2003;17:463‐471. 14730800 10.1108/14777260310506614

[7] Wood M , Ferlie E , Fitzgerald L . Achieving clinical behaviour change: a case of becoming indeterminate. 1998; 47:1729–1738. 10.1016/s0277-9536(98)00250-0 9877343

[8] Serres M . The Parasite. Baltimore and London: The John Hopkins University Press; 1982.

[9] Evidence‐Based Medicine Working Group . Evidence‐based medicine: a new approach to teaching the practice of medicine. JAMA. 1992;268:2420‐2425. 1404801 10.1001/jama.1992.03490170092032

[10] Greenhalgh T , Russell J . Why do evaluations of eHealth programs fail? An alternative set of guiding principles. PLoS Med. 2010;7:1‐5. 10.1371/journal.pmed.1000360 PMC2970573 21072245

[11] Catwell L , Sheikh A . Evaluating eHealth interventions: the need for continuous systemic evaluation. PLoS Med. 2009;6:1‐6. 10.1371/journal.pmed.1000126 PMC2719100 19688038

[12] Sackett D . Evidence based medicine: what it is and what it isn't. BMJ Br Med J. 1996;72:71‐72. 10.1136/bmj.312.7023.71 PMC2349778 8555924

[13] Kelly MP , Heath I , Howick J , Greenhalgh T . The importance of values in evidence‐based medicine. BMC Med Ethics. 2015;16:69. 26459219 10.1186/s12910-015-0063-3 PMC4603687

[14] Gøtzsche PC , Jørgensen KJ . Screening for breast cancer with mammography In: Gøtzsche PC, Chichester UK, eds. Cochrane Database Syst Rev. John Wiley & Sons, Ltd; 2013:CD001877. 10.1002/14651858.CD001877.pub5 PMC6464778 23737396

[15] Godlee F , Groves T . The new BMJ policy on sharing data from drug and. 2013, 7888(November 2012):1–3. 10.1136/bmj.e7888 23169872

[16] Jefferson T , Doshi P . Multisystem failure: the story of anti‐influenza drugs. BMJ. 2014;348: (apr10 14)g2263‐g2263. 24721793 10.1136/bmj.g2263

[17] Le Noury J , Nardo JM , Healy D , et al. Study 329 continuation phase: safety and efficacy of paroxetine and imipramine in extended treatment of adolescent major depression. Int J Risk Saf Med. 2016;28:143‐161. 27662279 10.3233/JRS-160728 PMC5044781

[18] Tsoukas H . The tyranny of light. Futures. 1997;29:827‐843.

[19] Green J . Epistemology, evidence and experience: evidence based health care in the work of Accident Alliances. Sociol Health Illn. 2000;22:453‐476.

[20] Loder E , Groves T . The BMJ requires data sharing on request for all trials. BMJ. 2015;350:h2373. 25953153 10.1136/bmj.h2373

[21] AllTrials: *All Trials Declaration* . 2013(September):1–8.

[22] Edwards A , Elwyn G . Shared decision‐making in health care: achieving evidence‐based patient choice. 2009:3–10.

[23] Ekman I , Swedberg K , Taft C , et al. Person‐centered care—ready for prime time. Eur J Cardiovasc Nurs. 2011;10:248‐251. 21764386 10.1016/j.ejcnurse.2011.06.008

[24] Vandvik PO , Otto CM , Siemieniuk RA , et al. Transcatheter or surgical aortic valve replacement for patients with severe, symptomatic, aortic stenosis at low to intermediate surgical risk: a clinical practice guideline. BMJ. 2016;5085(September):i5085. 10.1136/bmj.i5085 27680583

[25] Elwyn G , Quinlan C , Mulley A , Agoritsas T , Vandvik PO , Guyatt G . Trustworthy guidelines—excellent; customized care tools—even better. BMC Med. 2015;13:199. 26324120 10.1186/s12916-015-0436-y PMC4556022

[26] Mol A . The Logic of Care: Health and the Problem of Patient Choice. Routledge; 2008.

[27] Schei E . Doctoring as leadership. The power to heal. Perspect Biol Med. 2006;49:393‐406. 16960309 10.1353/pbm.2006.0048

[28] Hacking I . An Introduction to Probability and Inductive Logic. Cambridge university press; 2001.

[29] Gigerenzer G , Brighton H . Homo heuristicus: why biased minds make better inferences. Top Cogn Sci. 2009;1:107‐143. 25164802 10.1111/j.1756-8765.2008.01006.x

[30] Stolper E , Van De Wiel M , Van Royen P , Van Bokhoven M , Van Der Weijden T , Dinant GJ . Gut feelings as a third track in general practitioners' diagnostic reasoning. J Gen Intern Med. 2011;26:197‐203. 20967509 10.1007/s11606-010-1524-5 PMC3019314

[31] Gabbay J , Le May A . Evidence based guidelines or collectively constructed “mindlines?” Ethnographic study of knowledge management in primary care. BMJ Br Med J. 2004;329:1013. 15514347 10.1136/bmj.329.7473.1013 PMC524553

[32] Zwolsman SE , van Dijk N , de Waard MW . Observations of evidence‐based medicine in general practice. Perspect Med Educ. 2013;2:196‐208. 24002687 10.1007/s40037-013-0078-8 PMC3792233

[33] Mmath KET , Zwarenstein M , Oxman AD , et al. Analysis a pragmatic—explanatory continuum indicator summary ( PRECIS ): a tool to help trial designers. Can Med Assoc J. 2009;180. 10.1503/cmaj.090523 PMC2679824 19372436

[34] Zuiderent‐Jerak T , Forland F , Macbeth F . Guidelines should reflect all knowledge, not just clinical trials. BMJ. 2012;345(October):e6702. 10.1136/bmj.e6702 23043093

[35] Engebretsen E , Heggen K , Wieringa S , Greenhalgh T . Uncertainty and objectivity in clinical decision making: a clinical case in emergency medicine. Med Health Care Philos. 2016. 10.1007/s11019-016-9714-5 PMC5088215 27260370

[36] Contandriopoulos D , Lemire M , Denis J‐L , Tremblay E . Knowledge exchange processes in organizations and policy arenas: a narrative systematic review of the literature. Milbank Q. 2010;88:444‐483. 21166865 10.1111/j.1468-0009.2010.00608.x PMC3037172

[37] Staley K , Doherty C . It's not evidence, it's insight: bringing patients' perspectives into health technology appraisal at NICE. Res Involv Engagem. 2016;1–12. 10.1186/s40900-016-0018-y PMC5611625 29062505

[38] Russell J , Greenhalgh T . Being “rational” and being “human”: how National Health Service rationing decisions are constructed as rational by resource allocation panels. Health (London). 2013;18:441‐457. 24287296 10.1177/1363459313507586

[39] Jonsen AR , Toulmin S . The Abuse of Casuistry. Reprint ed. (1 July 1992) University of California Press; 1992.

[40] Kerry R , Eriksen TE , Lie SAN , Mumford SD , Anjum RL . Causation and evidence‐based practice: an ontological review. J Eval Clin Pract. 2012;18:1006‐1012. 22994999 10.1111/j.1365-2753.2012.01908.x

[41] Tonelli MR . The philosophical limits of evidence‐based medicine. Acad Med. 1998;73:1234‐1240. 9883197 10.1097/00001888-199812000-00011

[42] Askheim C , Sandset T , Engebretsen E . Who cares? The lost legacy of Archie Cochrane. Med Humanit. 2016;medhum‐2016‐011037. 10.1136/medhum-2016-011037 28228571

